# When Victims Become Abusers: A Study Among the Male Victims of Child Sexual Abuse in Bangladesh

**DOI:** 10.1177/08862605251318280

**Published:** 2025-02-12

**Authors:** Md. Abu Bakkar Siddik, Md. Rajwanullha Shakil, Monia Manjur, Md. Ishtiaq Ahmed Talukder, Md. Bashir Uddin Khan, Muhammad Asadullah

**Affiliations:** 1 Nanjing University, Nanjing, Jiangsu, China; 2 The Center for Social Policy and Justice, Dhaka, Bangladesh; 3 Hajee Mohammad Danesh Science and Technology University, Dinajpur, Bangladesh; 4 Noakhali Science and Technology University, Bangladesh; 5 Mawlana Bhashani Science and Technology University, Santosh, Tangail, Bangladesh; 6 University of Regina, Saskatchewan, SK, Canada

**Keywords:** child sexual abuse, male survivors, sexually offensive behaviors, suicidal ideation, repetitive abuse

## Abstract

There are substantial mental health consequences for male child sexual abuse (MCSA) victims. Survivors may exhibit sexually offensive actions because of this trauma. In other words, the abused becomes an abuser. In Bangladesh, MCSA is an invisible social problem. This study aimed to assess sexually offensive behaviors among victims of MCSA and to determine the associated factors. A total of 540 victims participated in an online survey as part of a cross-sectional study. Data were collected on victimization, suicidal ideation, history of offense, and sociodemographic factors. Pearson chi-square test and a binary logistic regression were employed to assess significant factors. Results revealed that 63.2% of participants reported engaging in sexually offensive behavior. Those living in villages, unmarried, experienced repetitive sexual abuse, under 13 years old at the time of abuse, experienced physical abuse concurrently, being penetrated during abuse, not disclosing the abuse, not receiving psychological assistance, having significant sexual involvement with men, and experiencing suicidal ideation were more likely to exhibit sexually offensive behaviors. The study underscores the importance of policymakers implementing relevant policies to safeguard boys. In addition, it emphasizes the need for victims to disclose instances of sexual abuse and actively seek psychological intervention.

## Introduction

Sexual abuse broadly refers to any unwanted sexual act, including noncontact offenses like exhibitionism or voyeurism, contact offenses such as unwanted kissing or intercourse, and exploitation for gain, such as taking nonconsensual nude photos or coercing someone into sexual activities. The global consciousness surrounding the alarming potential for children to fall victim to sexual predators has undeniably reshaped familial dynamics and societal attitudes. Often, perpetrators of such heinous acts are identified as male adults or adolescents who maintain proximity to the child, often within the same household (Långström et al., 2000; Ryan & Otonichar, 2016). It was estimated that the prevalence rates of child sexual abuse (CSA) for females varied anywhere from 8% to 31%, while the prevalence rates for boys ranged anywhere from 3% to 17% (Barth et al., 2013). Children who were victims of nonsexual abuse, sexual abuse without physical contact, sexual abuse without penetration, and sexual abuse with physical contact were all studied in the same large population research. Severe sexual abuse was linked to adult psychopathology and socioeconomic consequences (Alanko et al., 2017). Many negative health and social effects, such as self-harm, mental illnesses, and even physical health diagnoses like HIV and obesity, have been linked to sexual abuse in children (Amado et al., 2015; Arriola et al., 2005; Chen et al., 2010; Danese & Tan, 2013; Jespersen et al., 2009).

The most mentioned risk factors for individuals who become abusers later in life are being male and having experienced sexual abuse during childhood. Many professionals who work with children who have been sexually abused believe that individuals who perpetrate abuse were often victims of abuse themselves during childhood (Drury et al., 2019; Gekoski et al., 2016; Långström et al., 2000; Santhosh, 2016).

A longitudinal study conducted on males who experienced sexual abuse revealed that out of a total of 224 individuals, 26 were identified as victim abusers, having subsequently engaged in sexual offenses (Salter et al., 2003). Research by Levenson et al. (2014a) indicated that sex offenders were more than three times as likely to have experienced CSA as men from the general community. Another study examining female sex offenders found that approximately 50% of them reported experiencing childhood sexual abuse, a rate significantly higher than that observed in the general population. Half of the sexual offenders had a childhood sexual abuse history (Levenson et al., 2014a). Furthermore, an additional study revealed that individuals who engage in CSA reported a higher frequency of past experiences involving CSA (Simons et al., 2008). In addition, it has been noted by researchers that childhood sexual abuse is identified as a distinct developmental risk indicator for engaging in sexual abuse against children (Lee et al., 2002).

Numerous scholars have put forth the proposition that there exists a distinct correlation between the occurrence of childhood sexual abuse and the `engagement in sexual offenses during adulthood, commonly referred to as the sexually abused–sexual abuser hypothesis (Seto, 2008; Ward et al., 2008). Forensic examinations of adult and juvenile sex and non-sex offender samples provide the most compelling evidence for the sexually abused-abuser theory. According to two major meta-analyses, the incidence of sexual abuse among sex offenders is 3.4 and 2.8 times greater among sex offenders than non-sex offenders (Jespersen et al., 2009; Seto & Lalumière, 2010).

The main theme of this hypothesis is that most of the CSAs had a previous history of childhood sexual abuse (Carpentier & Proulx, 2011; Dennison & Leclerc, 2011). Several studies have researched the “sexually abused abuser hypothesis” using population-based samples. Three surveys conducted in the United States among high school students provide empirical evidence supporting the association between exposure to CSA and the manifestation of sexual aggression in the targeted population (Borowsky et al., 1997; Casey et al., 2008; Lodico et al., 1996).

While there is some evidence from population studies that supports the “sexually abused abuser hypothesis,” specifically about male youths, it is important to note that research is scarce within the academic community in Bangladesh concerning male survivors of CSA. This scarcity of research on such a delicate subject matter is noteworthy. Moreover, discussions about male child sexual abuse (MCSA) are often stigmatized within certain cultural contexts, contributing to a pervasive sense of shame surrounding this topic. Among male survivors of child abuse in Bangladesh, recent research indicated that 67.21% were suffering from severe depression and 21.31% had tried suicide on some occasion (Siddik et al., 2024). Based on the researchers’ current understanding, our study represents the inaugural investigation into the phenomenon of sexually abusive behavior within the Bangladeshi context. The objective of this study is to determine the prevalence of individuals who transition from being victims of sexual abuse to becoming either new victims or perpetrators themselves, as well as to identify associated factors.

## Methodology

### Ethical Approval

The study project, with the identifier NSTU/SCI/EC/2023/174, was given by the Institutional Review Board at Noakhali Science & Technology University in Bangladesh. Before any online data was collected, all participants handed over their informed written consent online. Participants in the study all gave their informed permission.

### Study Design and Data Collection

CSA remains a taboo topic in Bangladesh due to prevailing cultural circumstances (Sultana et al., 2022). Hence, in an offline context, the collection of data becomes an unattainable endeavor. In a cross-sectional study from June to December 2023, a survey was conducted in which a total of 540 individuals participated across Bangladesh. These participants completed an online questionnaire in the form of a Google form, which included variables related to sociodemographics, suicidal ideation, sexual abuse history, and sexual offending measures. The participants were requested to indicate their willingness to partake in the study as a component of the written consent process.

### Inclusion and Exclusion Criteria

As inclusion criteria in this research, the following conditions were required: (a) Persons who were born in Bangladesh and are citizens of Bangladesh; (b) boys who were abused sexually as children by male perpetrators; (c) individuals who are more than 18 years old; and (d) individuals who provided their permission to take part in this research. Some of the rationales for exclusion were as follows: (a) Persons who were less than 18 years old; (b) boys who had been abused sexually by female offenders; and (c) individuals who had not consented to take part in the research.

### Measures

#### Sociodemographic and Related Measures

This study considered the following items for measuring the sociodemographic characteristics of the respondents: Age; occupation (student, working); living area (city, village); marital status (married, unmarried); age when abused (below 13, 14–19, more than 20); repetitive sexual abuse (yes, no); physical abuse while sexually abused (yes, no); sexual abuse type (external touch, penetration); relationship with the perpetrator while being abused (relatives, peers, strangers, others); disclosure (yes, no); received any kind of psychological treatment (yes, no); major sexual involvement (with man, with woman, both); relationship with the victim while perpetrating (relatives, peers, stranger, and others).

#### Suicidal Ideation

The Ask Suicide-Screening Questions is a brief and validated instrument to assess suicidal ideation among youth (Horowitz et al., 2012). This study used the tool validated by the National Institute of Mental Health. It has four questions regarding suicidal ideation such as “In the past few weeks, have you wished you were dead?” and “In the past week, have you been having thoughts about killing yourself?” with two answers, yes or no. If a participant responds “yes” to any of the four questions, then he is identified as having suicidal ideation (National Institute of Mental Health, n.d.).

#### Sexual Abuse Questionnaire

We used the questionnaire that was developed and validated by Mohler-Kuo et al. (2014) to determine whether the participants had experienced any sexual abuse while they were children (Mohler-Kuo et al., 2014). In their study, Rojas and Olver (2020) examined the validity and reliability of the Violence Risk Scale–Youth Sexual Offense Version (VRS-YSO), a tool used for assessing the risk of sexual offenses in youth and planning appropriate treatment strategies. The VRS-YSO, developed by Mohler-Kuo et al. (2014), was used in this study to assess static risk factors and traits. For this cross-sectional study, a standardized digital version of the VRS-YSO was adapted for secure use on WhatsApp and Facebook. A pilot survey with 23 participants confirmed the feasibility and reliability of this online format. The tool’s established psychometric properties include high inter-rater reliability (Intraclass Correlation Coefficient = .85) and strong predictive validity, with an AUC of 0.79 for predicting sexual recidivism, further supporting its use.

### Analysis

The data was compiled using Microsoft Excel. The analysis was conducted using SPSS 26.0 software after the data had been edited, cleaned, and formatted. The correlation matrix was used to analyze the multicollinearity of the dataset. Data dependency, multivariate normality, independence, and multicollinearity were all verified using the Kolmogorov-Smirnov test. A frequency table was employed to depict the variations in socioeconomic status. The Pearson chi-square test was employed to identify the significant variables associated with the independent variable. Later, a binary logistic regression model was utilized to analyze the proportions of factors.

## Results

### Sociodemographic Profile of the Participants

Table 1 shows the demographic information of the research participants. With an average age of 21.3 years, 540 respondents joined the survey. 62.9% of the participants were students, and 37% were involved with different works. In terms of living area, 60.1% were living in the city, and 39.8% were from village areas. Among the respondents, 33.3% were married, while 66.6% were unmarried.

**Table 1.** Sociodemographic Characteristics and Reported Involvement in Offending Behavior.

Variables	Frequency (%), *n* = 540	Yes	No	*p*-value
		Count (*N* %)	Count (*N* %)	
Age	Mean Age 21.6 years old			
Occupation				
Student	340 (62.9)	76.2	23.8	.981
Working	200 (37.0)	30.1	69.9	
Living area				
City	325 (60.1)	25.5	74.5	.001**
Village	215 (39.8)	67.7	32.3	
Marital status				
Married	180 (33.3)	20.9	79.1	.05*
Unmarried	360 (66.6)	89.6	10.4	
Age when abused				
Below 13	314 (58.1)	61.1	38.9	.003*
14–19	134 (24.8)	47.4	52.6	
More than 20	92 (17.0)	13.7	86.3	
Repetitive sexual abuse				
No	159 (29.4)	33.3	66.7	.001**
Yes	381 (70.5)	89.3	10.7	
Physical abuse while sexually abused				
No	211 (39.0)	27.8	72.2	.001**
Yes	329 (60.9)	61.6	38.4	
Sexual abuse type				
External touch	123 (22.7)	39.4	60.6	.03*
Penetration	417 (77.2)	72.1	27.9	
Relationship with the perpetrator while being abused				
Relatives	433 (80.1)	65.2	34.8	.01*
Peers	51 (9.4)	50.4	49.6	
Stranger	34 (6.2)	43.0	57.0	
Others	22 (4.0)	52.5	47.5	
Disclosure				
No	482 (89.2)	78.8	21.2	.05*
Yes	58 (10.7)	11.7	88.3	
Received any kind of psychological treatment				
No	521 (96.4)	69.9	30.1	.001**
Yes	19 (3.5)	5.3	94.7	
Major sexual involvement				
With man	437 (80.9)	93.4	6.6	.003*
With woman	21 (3.8)	21.5	78.5	
Both	82 (15.1)	50.3	49.7	
Relationship with the victim while perpetrating				
Relatives	403 (74.6)	87.8	12.2	.552
Peers	79 (14.6)	52.7	47.3	
Stranger	44 (8.1)	37.3	100	
Others	14 (2.5)	48.6	51.4	
Suicidal ideation				
No	163 (30.1)	31.1	68.9	.02*
Yes	377 (69.8)	87.2	12.8	

* *p*-Value significant level ≤.05.

** *p*-Value significant level ≤.001.

### Prevalence of Offending

**Figure 1.**
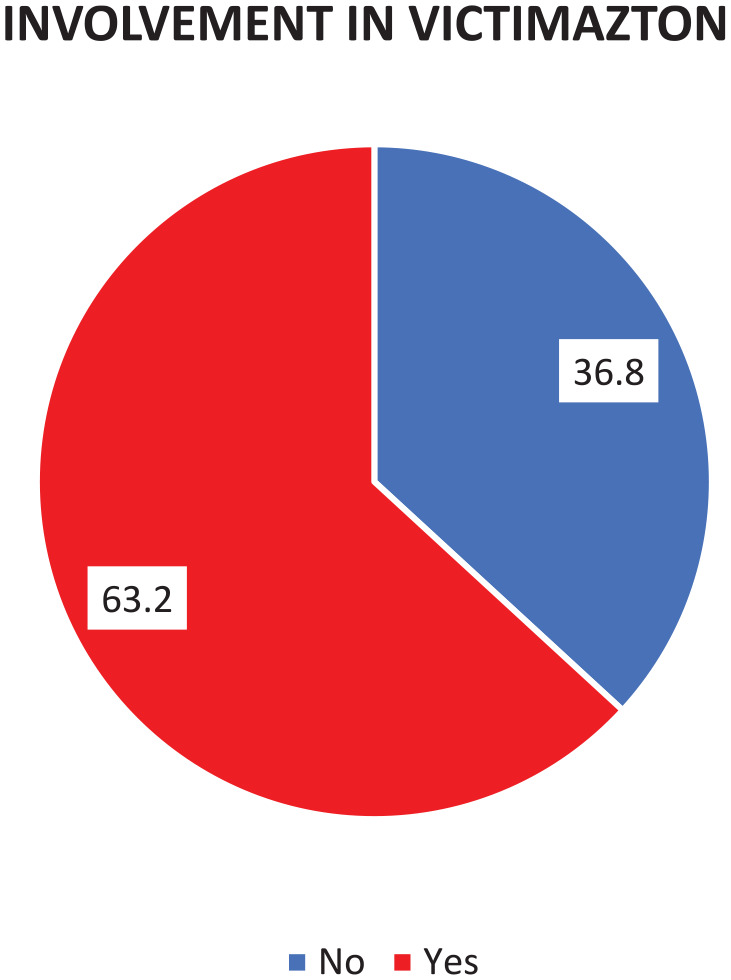
Involvement in offending.

The prevalence of involvement in victimization among the survivors is seen in Figure 1. Surprisingly, the study discovered that 63.2% of survivors reported having their involvement. Additionally, Figure 1 demonstrated that 36.8% of respondents admitted to involvement in offending behavior, while others denied any such involvement.

### Factors Associated with Offending Among the Participants

From Table 1, the data analysis does not reveal any specific trend or association between occupation and involvement in victimizing others. However, living area (*p* = .001) showed a significant effect on individuals becoming offenders, as 67.7% of respondents from villages (39.8%, *N* = 540) reported “yes” in this circumstance, compared to respondents from the city (60.1%, *n* = 540). The unmarried respondents (66.6%, *N* = 540) were significantly higher and involved (89.6%) in offending than the married respondents (33.3%, *N* = 540). Relationship of the respondent’s age when abused (*p* = .003), repetitive sexual abuse (*p* = .001), physical abuse while sexually abused (*p* = .001), and types of sexual abuse (*p* = .03) with the involvement in making other victims are statistically significant. Respondents below the age of 13 (58.1%, *N* = 540) and who were the victim of repetitive sexual abuse (70.5%, *N* = 540) were at a high risk of involving in offending. Table 1 also demonstrates that the respondent’s relationship with the perpetrator while being abused (*p* = .01) is highly statistically significant. Participants shared that they had not had a disclosure (89.2%, *N* = 540) at the higher-risk zone. Maximum respondents expressed not getting any psychological treatment (96.4%, *N* = 540) presenting higher involvement (69.9%), and depicted that the result is highly statistically significant (*p* = .001). Major sexual involvement (*p* = .003) and suicidal ideation (*p* = .02) had a significant effect on the involvement in victimizing others, while the relationship with the victim while perpetrating is not statistically significant.

**Figure 2.**
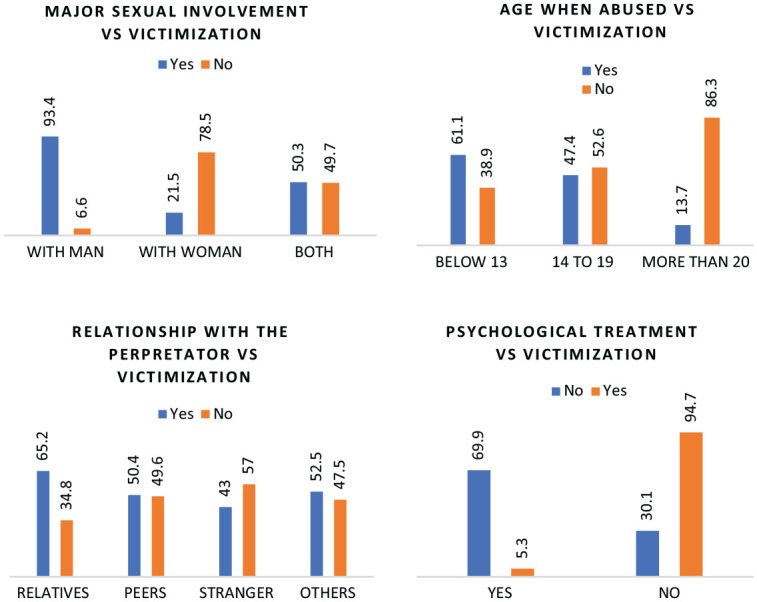
Different significant factors vs victimization.

Figure 2 illustrates the relationship between victimization and several significant factors. Individuals with male partners report the highest victimization rates, while age below 13 also correlates strongly with victimization. Relatives are the most common perpetrators, and a lack of psychological treatment is prevalent among victims. These insights highlight critical patterns in victimization based on sexual involvement, age, perpetrator relationship, and treatment access.

### Logistic Regression Analysis

Binary logistic regression in Table 2 represents the factors behind the involvement in victimizing others. It shows that occupation and relationship with the victim while perpetrating have no significant association with victimizing others. On the other hand, it presents that, participants from village areas (OR = 2.35; 95% CI [1.53, 3.60]), unmarried (OR = 1.56 [0.73, 3.32]), respondents who were abused before the age of 13 (OR = 2.35 [1.53, 3.60]) and 14 to 19 (OR = 1.87 [1.20, 2.90]) chronologically more likely at risk than the respondents from the city, married and respondents aged more than 20.

**Table 2.** Factors Associated with Involvement in Victimizing Others.

Variables	Exp (*B*)	95% CI [Lower, Upper]
Age	Mean age 21.6 years old	Occupation
Student		1.21 [0.85, 1.71]
Working	Reference	
Living area		
Village		2.35 [1.53, 3.60]
City	Reference	
Marital status		
Unmarried		1.56 [0.73, 3.32]
Married	Reference	
Age when abused		
Below 13		2.35 [1.53, 3.60]
14–19		1.87 [1.20, 2.90]
More than 20	Reference	
Repetitive sexual abuse		
Yes		4.67 [3.19, 6.85]
No	Reference	
Physical abuse while sexually abused		
Yes		3.96 [2.72, 5.76]
No	Reference	
Sexual abuse type		
Penetration		4.41 [2.90, 6.72]
External touch	Reference	
Relationship with the perpetrator while being abused		
Relatives		3.38 [2.56, 4.45]
Peers		2.47 [1.91, 3.19]
Stranger		1.26 [0.98, 1.63]
Others	Reference	
Disclosure		
No		3.87 [2.78, 5.40]
Yes	Reference	
Received any kind of psychological treatment		
No		6.65 [4.76, 9.29]
Yes	Reference	
Major sexual involvement		
With man		4.64 [2.72, 7.91]
Both		2.56 [1.76, 3.73]
With woman	Reference	
Relationship with the victim while perpetrating		
Relatives		1.67 [1.16, 2.41]
Peers		1.46 [1.06, 2.01]
Stranger		1.56 [0.73, 3.32]
Others	Reference	
Suicidal ideation		
Yes		3.66 [2.30, 5.82]
No	Reference	

*Note.* CI = confidence interval.

Respondents who experienced repetitive sexual abuse (OR = 4.67; 95% CI [3.19, 6.85]) and who had gone through sexual abuse associated with physical abuse (OR = 3.96 [2.72, 5.76]) were more likely to be involved in victimizing others. Those who were penetrated (OR = 4.41 [2.90, 6.72]) during sexual abuse are 4.41 times more likely to be involved in the offending process, regardless of the type of abuse. Those who were abused by peers (OR = 2.47 [1.91, 3.19]) and strangers (OR = 1.26 [0.98, 1.63]) were more likely to be at risk of becoming offenders. The victims who did not disclose their abuse were 3.8 times (OR = 3.87 [2.78, 5.40]) more likely to have offending behavior than those who disclosed their abuse history.

Participants who were involved in major sexual involvement with men (OR = 4.64; 95% CI [2.72, 7.91]) and with both genders (OR = 2.56 [1.76, 3.73]) were more likely to be in danger than those who were involved with women. Participants expressed those persons with suicidal ideation 3.6 times (OR = 3.66 [2.30, 5.82]) are more likely to be involved in victimizing others. Table 2 also demonstrates that persons who did not receive any form of psychological treatment (OR = 6.65 [4.76, 9.29]) are more likely to be involved in victimizing others.

## Discussion

Research indicates that the estimated prevalence of CSA ranges from 8% to 31% for females and from 3% to 17% for males (Barth et al., 2013). This study also explored sexually offensive behavior among MCSA survivors and assessed the significant factors associated with the offending. We found that factors such as living area, marital status, age at the time of the abuse, repetitive sexual abuse, physical abuse during sexual abuse, type of sexual abuse, relationship with the perpetrator during the abuse, disclosure, receipt of any kind of psychological treatment, major sexual involvement, and relationship with the victim while perpetrating were significantly associated with offending behaviors.

Following prior research and theoretical assumptions, individuals who have experienced CSA were found to have a higher likelihood of self-reporting engaging in coercive sexual behaviors toward others, compared to individuals who have not experienced CSA (Borowsky et al., 1997; Casey et al., 2008; Seto & Lalumière, 2010). Research conducted among Swiss males revealed that the occurrence of sexual abuse was determined to be of comparable significance to other risk factors for the development of sexually abusive behaviors in male adolescents (Aebi et al., 2015). Similar findings were reported by Seto & Lalumière (2010) in samples of male teenagers from Norway and Sweden; those with a history of CSA were more likely to report coercive sexual behaviors, even after adjusting for other sexual behaviors (such as pornography usage), drug abuse, and nonsexual violent behavior. Our study also identified similar results. For instance, 63.1% of sexually abused males abused another individual.

The prevalence of CSA tends to vary across different areas. Cities reported a greater incidence of sexual abuse as compared to those living in rural regions; those living in rural areas reported a lower frequency (Karayianni et al., 2017; Scheper-Hughes & Devine, 2003). In a separate study, it was observed that survivors of CSA from urban areas reported a higher frequency of CSA incidents compared to those from townships or rural regions (Feng et al., 2010). In addition, research has suggested that the likelihood of becoming an offender is higher in city areas (Becker et al., 1986). Our research found that individuals residing in rural areas exhibited a higher prevalence of engaging in offensive behavior as compared to those residing in cities. This aspect presents a notable departure from the findings of previous research. Due to the conservative cultural norms prevalent in rural areas, individuals may be hesitant to disclose incidents of abuse, creating opportunities for abusers to victimize others.

The drive to have sexual encounters is one of the defining characteristics of the human species (Ainsworth, 2015). In this scenario, it appears that men who are not married or in committed relationships exhibit a higher level of sexual desire (Yuk-ha Tsang, 2019). The study found a positive correlation between being unmarried or unattached and an increased likelihood of engaging in sexual offending behavior (Olver & Wong, 2017). Numerous other studies examining sexual offenders have indicated that married individuals who had been sexually abused in childhood exhibit a lower propensity for reoffending compared to their unmarried counterparts (Hilarski et al., 2008; McGrath, 2013; Rice et al., 2013). One of the key findings of this study supported the previous findings that unmarried survivors were at higher risk of offending.

There is evidence to suggest that individuals who have experienced multiple instances of sexual abuse may be more susceptible to engaging in offending behaviors later in life (Levenson et al., 2014a). In a study conducted by Claudia et al. (2015), it was observed that individuals who experienced multiple forms of maltreatment were more likely to be associated with a higher prevalence of violent offenses (van der Put et al., 2015). The findings of several additional studies among male victims of CSA came to the same conclusion (Aebi et al., 2011, 2015; Levenson et al., 2014b; Nicholas Groth et al., 2016). The findings of our study aligned with those of the other studies. Repeated sexual abuse was another element that we discovered to be a significant predictor of becoming a sexual offender in later life.

The current research suggested that there is a higher likelihood of students engaging in sexual offenses against male children or adolescents compared to individuals who are currently employed. Nevertheless, the researchers encountered a paucity of studies about this discovery. Additional research would be beneficial to obtain robust evidence.

### Strengths and Limitations

To our knowledge, no previous studies in Bangladesh have specifically addressed the issue of survivors of MCSA. This study represents the first investigation into the sexual offending behavior exhibited by MCSA survivors, marking a significant strength of the research. Another strength is the study’s robust sample size, which enhances the generalizability of the findings. In addition, this work’s strongest aspect lies in identifying key accelerators for offensive conduct. However, the reliance on self-reported questionnaires is a notable limitation, as it may introduce biases. Furthermore, corrections for multiple analyses were not applied, which increases the risk of Type I errors and is acknowledged as a constraint. The research could also include other conspicuous variables for a more comprehensive analysis. Despite these limitations, this study provides valuable insights and sets a foundation for further exploration in this critical area.

## Conclusion

Individuals who are victims of sexual abuse by men often experience significant psychological repercussions as a result of the abuse. Among the survivors, the development of sexually offensive behavior is considered one of the most significant effects that might result from this trauma. This indicates that those who have been mistreated may become abusers. According to the findings of this research, a sizable percentage of the people who had been subjected to sexual abuse exhibited sexually objectionable conduct. We recommend that those responsible for formulating policies do so to break the vicious cycle of abuse. In addition, the victims need to attempt to access psychological assistance. It is recommended that people who have a significant amount of sexual interaction with other males obtain psychiatric therapy. Overall, it is necessary to put an end to the cycle of victimization.
